# Region-Specific Neurovascular Decoupling Associated With Cognitive Decline in Parkinson’s Disease

**DOI:** 10.3389/fnagi.2021.770528

**Published:** 2021-11-15

**Authors:** Song’an Shang, Hongying Zhang, Yuan Feng, Jingtao Wu, Weiqiang Dou, Yu-Chen Chen, Xindao Yin

**Affiliations:** ^1^Department of Radiology, Nanjing First Hospital, Nanjing Medical University, Nanjing, China; ^2^Department of Radiology, Clinical Medical College, Yangzhou University, Yangzhou, China; ^3^MR Research China, GE Healthcare, Beijing, China

**Keywords:** Parkinson’s disease, neurovascular coupling, cognitive impairment, magnetic resonance imaging, regional homogeneity, cerebral blood flow

## Abstract

**Background:** Cognitive deficits are prominent non-motor symptoms in Parkinson’s disease (PD) and have been shown to involve the neurovascular unit (NVU). However, there is a lack of sufficient neuroimaging research on the associated modulating mechanisms. The objective of this study was to identify the contribution of neurovascular decoupling to the pathogenesis of cognitive decline in PD.

**Methods:** Regional homogeneity (ReHo), a measure of neuronal activity, and cerebral blood flow (CBF), a measure of vascular responses, were obtained from patients with PD with mild cognitive impairment (MCI) and normal cognition (NC) as well as matched healthy controls (HCs). Imaging metrics of neurovascular coupling (global and regional CBF-ReHo correlation coefficients and CBF-ReHo ratios) were compared among the groups.

**Results:** Neurovascular coupling was impaired in patients with PD-MCI with a decreased global CBF-ReHo correlation coefficient relative to HC subjects (*P* < 0.05). Regional dysregulation was specific to the PD-MCI group and localized to the right middle frontal gyrus, right middle cingulate cortex, right middle occipital gyrus, right inferior parietal gyrus, right supramarginal gyrus, and right angular gyrus (*P* < 0.05). Compared with HC subjects, patients with PD-MCI showed higher CBF-ReHo ratios in the bilateral lingual gyri (LG), bilateral putamen, and left postcentral gyrus and lower CBF-ReHo ratios in the right superior temporal gyrus, bilateral middle temporal gyri, bilateral parahippocampal gyri, and right inferior frontal gyrus. Relative to the HC and PD-NC groups, the PD-MCI group showed an increased CBF-ReHo ratio in the left LG, which was correlated with poor visual–spatial performance (*r* = −0.36 and *P* = 0.014).

**Conclusion:** The involvement of neurovascular decoupling in cognitive impairment in PD is regionally specific and most prominent in the visual–spatial cortices, which could potentially provide a complementary understanding of the pathophysiological mechanisms underlying cognitive deficits in PD.

## Introduction

Parkinson’s disease (PD) is an age-related neurodegenerative disorder with primary pathological features of selective and progressive dopamine depletion and Lewy body deposition, accounting for clinical manifestations with cardinal motor disturbances as well as a spectrum of non-motor dysfunction (Kalia and Lang, 2015; Dickson, 2018). The investigation of cognitive impairment, a prevalent non-motor symptom, has been highlighted due to concerns about its early onset and the associated risk of progression to dementia (Aarsland et al., 2017; Baiano et al., 2020; Ryman and Poston, 2020). Notably, mild cognitive impairment in PD (PD-MCI) has been defined as a transitional stage between normal aging and dementia with a prevalence of 20–30% among those with PD (Baiano et al., 2020). In contrast with the well-characterized pathology in motor symptoms, the cognitive status in patients with PD-MCI deteriorates through the integration of pathological effects, including abnormal neurotransmitter systems (dopaminergic and cholinergic systems) (Olde Dubbelink et al., 2014) and aberrant protein aggregates (α-synuclein and amyloid-β) (Hepp et al., 2016). A comprehensive understanding of the pathophysiological mechanisms in PD-MCI is still needed.

Recent studies have suggested that neurovascular coupling, one of the pivotal mechanisms that maintain homeostasis in the cerebral microenvironment, is disrupted in several pathological conditions, such as AD (Solis et al., 2020), cerebrovascular diseases (Cai et al., 2017), and traumatic brain injury (Jang et al., 2017). In fact, as the primary components of the neurovascular unit (NVU), neurons and the vasculature could also be vulnerable to the involvement of neuroinflammation and oxidative stress in PD (Campolo et al., 2019). Emerging evidence based on a blood oxygen level-dependent (BOLD) approach has revealed that patients with PD-MCI experienced aberrant neuronal activity mainly in cortices in the default mode network (DMN) and motor network, which corresponded to symptomatic presentations (Amboni et al., 2015; Pan et al., 2017; Bezdicek et al., 2018). Meanwhile, cerebral blood flow (CBF) can be quantified as a non-invasive surrogate of metabolic demand by the arterial spin labeling (ASL) approach; such studies with patients with PD-MCI have shown a distribution of parietal–occipital hypoperfusion and a PD-related perfusion pattern (PDRP) that were analogous to radiotracer findings (Melzer et al., 2011; Arslan et al., 2020). However, there is still a paucity of neuroimaging investigations regarding neurovascular decoupling and the consequent cognitive deficits in PD-MCI.

As a functional complex, alterations in neuronal activity and cerebral perfusion in the NVU are interactive rather than independent (Phillips et al., 2016). Recently, a novel imaging metric derived from MRI techniques (BOLD and ASL) was introduced for the assessment of NVU function (Liang et al., 2013). Although some regions of neurovascular decoupling were shown to be involved in cognitive impairment in cerebrovascular disease (Liu et al., 2021), whether the heterogeneous cognitive profiles in PD-MCI are potentially driven by NVU dysregulation is inconclusive. Therefore, this study aimed to investigate neurovascular decoupling in PD-MCI by integrating regional homogeneity (ReHo) information, a measure of neuronal activity, and CBF information, a measure of cerebral perfusion. The intergroup differences in the CBF-ReHo coupling coefficient at the global and regional levels and CBF-ReHo ratios at a voxel-wise level were compared among patients with PD-MCI, patients with PD and normal cognition (PD-NC), and healthy controls (HCs). The correlations between neurovascular decoupling and clinical variables were further explored. We hypothesized that the responsible cortices would be more vulnerable to neurovascular decoupling, yielding potential imaging biomarkers for insight into the underlying pathological processes.

## Materials and Methods

### Patients

In total, 92 right-handed patients with PD from the clinic of movement disorders and 46 matched HC in terms of age, sex, handedness, and years of education from the local community were recruited from May 2019 to April 2021. This study was approved by the Research Ethics Committee of Nanjing Medical University, and written informed consent was obtained from all subjects 24 h before study entry. The Unified Parkinson’s Disease Rating Scale Part III (UPDRS-III) and Hoehn–Yahr (H-Y) scale were scored for disease severity and stage of PD, respectively. Levodopa equivalent daily dose (LEED) was also calculated in patients with PD. All subjects received neuropsychological assessments, including the Montreal Cognitive Assessment (MoCA), Hopkins Verbal Learning Test-Revised (HVLT-R), Symbol Digit Modalities Test (SDMT), Trail Making Test (TMT)-A and TMT-B, Boston Naming Test (BNT), and Benton’s Judgment of Line Orientation (JoLO), for the assessment of global cognition and five particular cognitive domains. Meanwhile, the Clinical Dementia Rating (CDR) was administered for the evaluation of dementia. All patients were instructed to withhold medication administration 12 h prior to clinical assessment and MRI data acquisition.

Patients were enrolled based on the following inclusion criteria: (1) patients met the United Kingdom Parkinson’s Disease Society Brain Bank Clinical Diagnostic Criteria (Hughes et al., 1992); (2) patients were at a disease stage within the H-Y stages I–III; (3) there was an absence of dementia based on the MDS diagnostic criteria for PD with dementia (Emre et al., 2007); and (4) antiparkinsonian medications with a stable and optimized daily dose were being administered for more than 4 weeks before enrollment. The HC subjects were required to be free of cognitive complaints and obtain scores in the normal range in the neuropsychological assessments. The exclusion criteria for all participants were as follows: (1) atypical or secondary parkinsonism; (2) other diseases involving the central neural system, including neurological or psychiatric diseases, cerebrovascular accident, trauma, and brain tumor; (3) history of alcoholism, smoking, any chronic disorders, or neurological surgery that affect cognitive status; (4) psychotropic or anticholinergic treatment; (5) excessive head motion (displacement >3 mm or rotation >3.0°) during the data preprocessing procedures; and (6) any MRI contraindication or severe hearing or visual loss. Subsequently, patients with PD who fulfilled the proposed Movement Disorders Society Task Force criteria for level I were included in the PD-MCI group, whereas other patients with PD were included in the PD-NC group. The demographic and clinical details are listed in Table 1.

**TABLE 1:** Demographic characteristics and scores of clinical assessments.

	HC (*n* = 46)	PD-NC (*n* = 47)	PD-MCI (*n* = 45)	*P*
Age	61.936.51	62.1710.27	63.119.39	0.80
Sex (M/F)	24/22	26/21	24/21	0.95
Education (years)	10.783.48	10.533.49	10.043.50	0.87
Disease duration (years)	N/A	3.552.48	3.762.33	0.57
UPDRS-III	N/A	28.6411.19	31.3610.41	0.25
H-Y stage	N/A	1.660.72	1.890.82	0.20
LEED	N/A	430.68146.75	454.04151.62	0.46
MoCA	28.201.19	27.641.49	21.162.28^a,b^	<0.001
HVLT-R (total)	21.873.99	20.774.26	18.623.58^a,b^	<0.001
HVLT-R (delayed)	7.802.21	7.152.48	6.512.42^a^	<0.001
HVLT-R (recognition)	8.672.16	8.262.26	7.732.03	0.12
SDMT	46.0411.61	42.4311.86	37.6713.08^a^	0.006
TMT-A	62.2015.61	68.0619.34	76.1117.57^a,b^	0.001
TMT-B	123.4331.43	137.9633.79	155.0243.30^a,b^	<0.001
BNT	24.723.23	23.173.61	18.985.17^a,b^	<0.001
JoLO	22.634.93	21.025.43	17.025.14^a,b^	<0.001

*Data were represented as mean ± SD; N/A indicates not applicable. ^a^Indicates significant difference to the HC group. ^b^Indicates significant difference to the PD-NC group.*

*HC, healthy control; PD, Parkinson’s disease; NC, normal cognition; MCI, mild cognitive impairment; M, male; F, female; UPDRS, Unified Parkinson’s Disease Rating Scale; H-Y, Hoehn–Yahr; LEED, levodopa equivalent daily dose; MoCA, Montreal Cognitive Assessment; HVLT, Hopkins Verbal Learning Test-Revised; SDMT, Symbol Digit Modalities Test; TMT, Trail Making Test; BNT, Boston Naming Test; JoLO, Benton’s Judgment of Line Orientation.*

### MRI Data Acquisition

MRI data for each individual were acquired using a 3.0-Tesla MRI scanner (Discovery MR750, GE Medical Systems, Milwaukee, WI, United States) with a commercial eight-channel phased-array head coil. During acquisition, all subjects wore headphones, were instructed to lie still in a supine position, and to stay awake with their eyes closed. Functional data of the whole brain were acquired using a gradient recalled echo echo-planar imaging sequence as follows: repetition time (TR), 2,000 ms; echo time (TE), 30 ms; flip angle (FA), 90°; slice thickness, 4 mm without gap; field of view (FOV), 240 mm × 240 mm; matrix size, 64 × 64; voxel size, 4.0 mm × 4.0 mm × 4.0 mm; number of time points, 240; each frame included 35 continuous slices that covered the whole brain volume; and total scan time, 480 s. Perfusion data were obtained using a background suppressed three-dimensional pseudocontinuous ASL (pCASL) sequence with the following parameters: TR, 10.5 ms; TE, 4.9 ms; FA, 111°; slice thickness, 4 mm without gap; FOV, 240 mm × 240 mm; matrix size, 128 × 128; labeling duration, 1,500 ms; postlabeling delay, 2,025 ms; number of excitations, 3; number of slices, 36; 8 arms with 512 points per arm; total scan time, 284 s; and units, ml/100 g/min. This sequence also included a fluid-suppressed proton density acquisition with the same image dimensions as the pCASL but without radio frequency labeling for CBF quantitation and image registration. Whole-brain structural images were also acquired using a three-dimensional high-resolution brain volume imaging sequence as follows: TR, 12 ms; TE, 5.1 ms; inversion time, 450 ms; FA, 15°; slice thickness, 1 mm with no gaps; FOV, 240 mm × 240 mm; matrix size, 256 × 256; voxel size, 1 mm × 1 mm × 1 mm; number of slices, 172; and total scan time, 320 s.

### Functional Data Preprocessing and Analysis

Data preprocessing was implemented using the Data Processing and Analysis for Brain Imaging toolkit (DPABI, version 4.6^1^) on the basis of statistical parametric mapping (SPM, version 12^2^) running on the MATLAB 2018a platform (MathWorks, Natick, MA, United States). In brief, the main preprocessing steps were as follows: removal of the initial 10 time points; slice timing of the remaining slices; realignment for head-motion correction by excluding subjects with excessive head movement (displacement >3 mm or rotation >3.0°); coregistration based on segmented T1 images; spatial normalization to Montreal Neurological Institute (MNI) space (resampling of 3 mm × 3 mm × 3 mm); linear detrending and temporal band-pass (0.01–0.08 Hz) filtering; and regression of nuisance covariates (i.e., Friston-24 parameters, global signals, white matter signals, and cerebrospinal fluid signals).

Regional homogeneity was applied to assess the regional signal synchronizations (Zang et al., 2004). For each individual, Kendall’s coefficient concordance (KCC) was calculated to measure the similarity between the time series of a functional voxel and its 26 neighboring voxels within a whole-brain mask. Finally, the voxel-wise ReHo map was standardized to a z-scored map by subtracting the global mean value of gray matter (GM) and then dividing by the SD and further spatially smoothed with a 6-mm full-width at half-maximum (FWHM) Gaussian kernel.

### Perfusion Data Preprocessing and Analysis

The CBF images for each subject were obtained using FuncTool software (Version 4.6, GE Medical Systems, Milwaukee, WI, United States) based on a general kinetic model for ASL (Ambarki et al., 2015) and were preprocessed using SPM12 running on the MATLAB 2018a platform. The main preprocessing procedures were as follows: motion and magnetic inhomogeneity correction by excluding subjects with excessive head movement (displacement >3 mm or rotation >3.0°); coregistration based on segmented T1 images and spatial normalization to MNI space (resampling of 3 mm × 3 mm × 3 mm); removal of non-brain tissues; z-scored standardization; and spatial smoothing with a 6-mm FWHM Gaussian kernel. Specifically, a relative increase in perfusion with respect to other groups was interpreted as preserved perfusion as previously described.

### Neurovascular Coupling Quantitative Analysis

The neurovascular coupling quantitative analysis was performed using the DPABI toolkit. The neurovascular coupling of each subject was quantitatively assessed by calculating the correlation coefficient between the z-ReHo map and z-CBF map across voxels at the whole-brain GM level (Zhu et al., 2017; Guo et al., 2019). Regional neurovascular coupling (Hu et al., 2019) was further analyzed by dividing the whole-brain GM into 90 independent regions based on the automated anatomical labeling (AAL) atlas (Rolls et al., 2020). Given that the spatial preprocessing (registration and spatial smoothing) would induce high dependence of neighboring voxels, the effective degree of freedom (df_eff_) in across-voxel correlation analyses would be much smaller than the number of voxels within the GM mask. Thus, we conducted the estimation for df_eff_ of across-voxel correlations with the following equation (Liang et al., 2013; Zhu et al., 2017):


$$d\,f_{e\,f\,f}=\frac{N}{\left(\mathrm{FWHMx}\times \text{FWHMy}\times \mathrm{FWHMz}\right)/\text{v}}-2$$

where *v* represents the voxel volume (3 mm × 3 mm × 3 mm), *N* represents the number of voxels used in the analyses (48,539), and FWHMx × FWHMy × FWHMz is the average smoothness of the CBF and ReHo maps (9.50 mm × 10.04 mm × 9.87 mm). In this study, df_*eff*_ of across-voxel correlations was 1,390. In addition, CBF-ReHo ratios (Liang et al., 2013), designed to reflect metabolic energy per unit of neuronal activity, across voxels within the GM mask were calculated with the original values of the metrics and subsequently *z*-scored for standardization.

### Statistical Analysis

The Kolmogorov–Smirnov test was employed to assess demographic and clinical data normality. Intergroup differences in sex distribution and age were analyzed using Chi-square tests or one-way analysis of variance (ANOVA), respectively. The UPDRS-III scores, H-Y stage, disease duration, and LEED between PD subgroups were compared using Mann–Whitney *U* tests. Kruskal–Wallis *H* tests (for years of education and MoCA scores) and ANOVAs (for scores of five cognitive domains, metrics of whole-brain GM, and regional neurovascular coupling) were utilized for comparisons among groups, followed by *post hoc* tests with Bonferroni correction. The above analyses were performed using SPSS 22.0 software (version 22.0, SPSS Inc., Chicago, IL, United States), and the significance level was set as a *P*-value < 0.05.

A voxel-wise one-way analysis of covariance within the GM mask was applied using SPM12 software for the investigations of differences (e.g., with CBF-ReHo ratios) among groups, followed by *post hoc* analysis within the significant clusters. The significance level was set at an uncorrected voxel-wise threshold of *P* < 0.001 and a familywise error (FWE)-corrected cluster-wise threshold of *P* < 0.05. The mean values of each cluster that showed significant intergroup differences were extracted. By using SPSS 19.0 software, the mean values of clusters (CBF-ReHo ratio, CBF values, and ReHo values) among three groups were compared using ANOVA, followed by *post hoc* test with Bonferroni correction; Pearson correlation tests (for normally distributed scores) or Spearman’s rank correlation tests (for non-normally distributed scores) were further applied to explore potential associations between values of these clusters and scores from the clinical assessments. A statistically significant threshold was set at a *P*-value < 0.05. The above analyses were adjusted for covariates, including age, sex, years of education, and GM volume (obtained from segmented T1 data).

## Results

### Demographic and Neuropsychological Characteristics

The demographic and neuropsychological characteristics of the three groups are summarized in Table 1. There were no significant differences in terms of age, sex, or education among the three groups or in disease duration, disease severity, H-Y stage, or LEED between the PD-NC and PD-MCI groups (*P* > 0.05). For the neuropsychological comparisons, patients with PD-MCI suffered from significant impairments within the global cognition and five domains compared with the other two groups (*P* < 0.05). None of the significant differences between the HC and PD-NC groups survived regarding global cognition or the five cognitive domains (*P* > 0.05).

### Alterations in Whole-Brain and Regional Cerebral Blood Flow-Regional Homogeneity Correlation Coefficients

**FIGURE 1:**
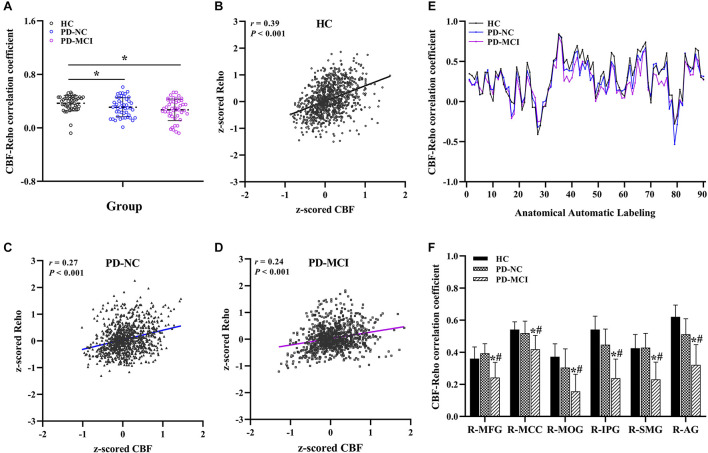
Global and regional neurovascular coupling among groups. The mean CBF-ReHo coupling at the global level among groups **(A)** and representative scatter plots of the spatial correlations across voxels in individuals **(B–D)**; regional neurovascular coupling of CBF-ReHo **(E)** and the significantly different brain regions in the PD-MCI group as compared with HC and PD-NC groups **(F)**. *Indicates significant intergroup difference to HC group; ^#^indicates significant intergroup difference to PD-NC group. CBF, cerebral blood flow; ReHo, regional homogeneity; HC, healthy control; PD, Parkinson’s disease; NC, normal cognition; MCI, mild cognitive impairment; R, right; L, left; MFG, middle frontal gyrus; MCC, middle cingulate cortex; MOG, middle occipital gyrus; IPG, inferior parietal gyrus; SMG, supramarginal gyrus; AG, angular gyrus.

Significant differences in the CBF-ReHo correlation coefficients at the whole-brain GM level were found among the groups (*P* = 0.005) (Figures 1A–D). Both the PD-MCI and PD-NC groups showed significantly reduced coefficients compared with the HC group (*P* = 0.001 and *P* = 0.044). Although no significant intergroup differences between the PD-NC and PD-MCI groups survived, a downward trend was observed among the groups. Regarding comparisons of the regional CBF-ReHo correlation coefficients, the HC group and PD-NC group showed significant differences relative to the PD-MCI group, which had lower coefficients in the right middle frontal gyrus (MFG), right middle cingulate cortex (MCC), right middle occipital gyrus (MOG), right inferior parietal gyrus (IPG), right supramarginal gyrus (SMG), and right angular gyrus (AG) (*P* < 0.05) (Figures 1E,F).

### Aberrant Distributions of Cerebral Blood Flow-Regional Homogeneity Ratios and Correlations

**FIGURE 2:**
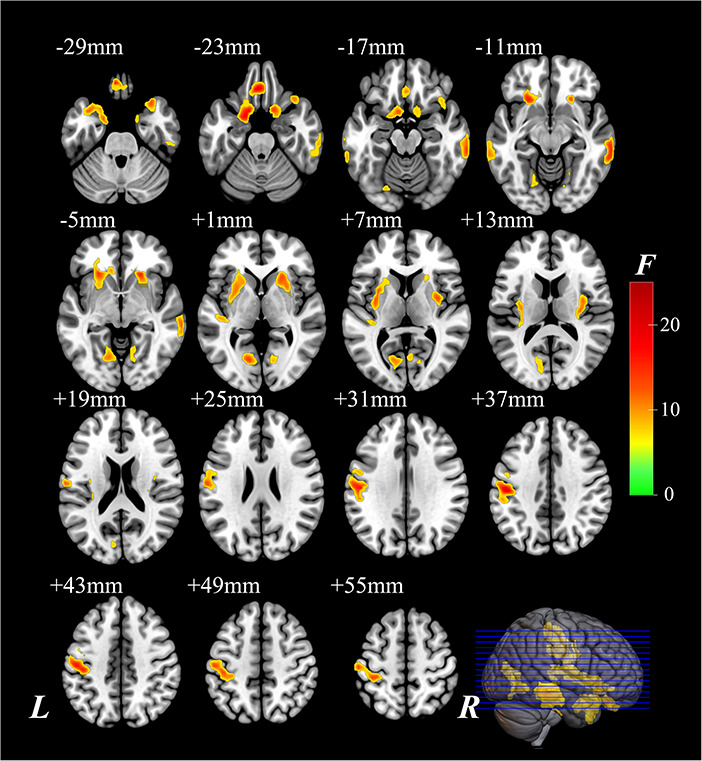
The brain regions with significant differences of the CBF-ReHo ratio among groups. The voxel-based analysis demonstrates the survived clusters among HC, PD-NC, and PD-MCI groups. These clusters are referred to multiple comparisons correction using the FWE rate (a cluster-defining threshold of *P* = 0.001 and a corrected cluster significance of *P* < 0.05). The region with the significant difference among the three groups is shown with warm color. CBF, cerebral blood flow; ReHo, regional homogeneity; HC, healthy control; PD, Parkinson’s disease; NC, normal cognition; MCI, mild cognitive impairment; FWE, familywise error; R, right; L, left.

**FIGURE 3:**
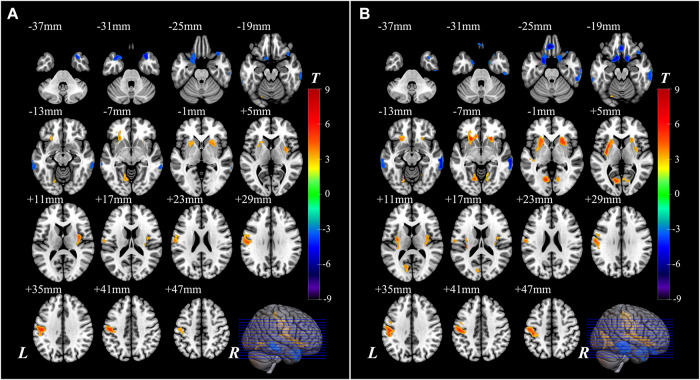
The brain regions with significant intergroup differences of the CBF-ReHo ratio. The voxel-based analysis demonstrates the survived clusters between the HC group and PD-NC group **(A)** and PD-MCI group **(B)**. These clusters are referred to multiple comparisons correction using the FWE rate (a cluster-defining threshold of *P* = 0.001 and a corrected cluster significance of *P* < 0.05). The significantly increased ratio relative to HC in the group is shown with warm color. The significantly decreased ratio relative to HC in the group is shown with cold color. CBF, cerebral blood flow; ReHo, regional homogeneity; HC, healthy control; PD, Parkinson’s disease; NC, normal cognition; MCI, mild cognitive impairment; FWE, familywise error; R, right; L, left.

**FIGURE 4:**
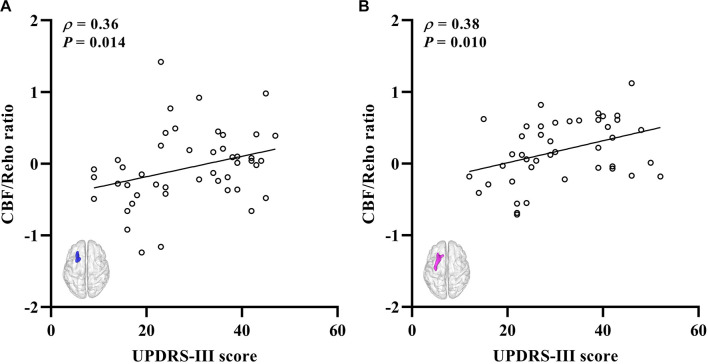
The correlations between the CBF-ReHo ratio and scores of disease severity. **(A)** Correlations for the PD-NC group between the scores of UPDRS-III (*X*-axis) and CBF-ReHo ratio in left putamen (*Y*-axis); **(B)** correlations for the PD-MCI group between the scores of UPDRS-III (*X*-axis) and CBF-ReHo ratio in left putamen (*Y*-axis). CBF, cerebral blood flow; ReHo, regional homogeneity; PD, Parkinson’s disease; NC, normal cognition; MCI, mild cognitive impairment; UPDRS, Unified Parkinson’s Disease Rating Scale.

**FIGURE 5:**
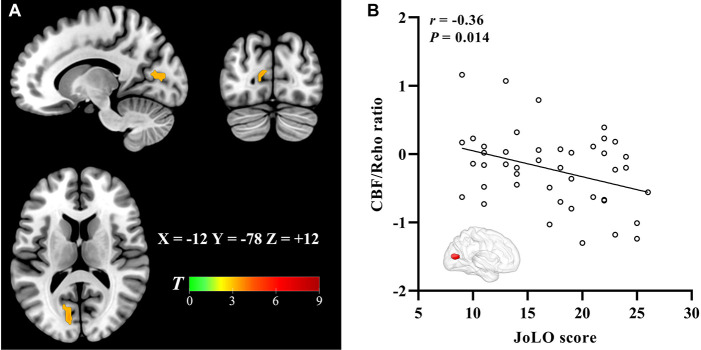
The brain regions with the significant CBF-ReHo ratio difference between PD-MCI and PD-NC groups and correlation to visual–spatial function. The voxel-based analysis demonstrates the survived clusters between PD-MCI and PD-NC groups **(A)**. These clusters are referred to multiple comparisons correction using the FWE rate (a cluster-defining threshold of *P* = 0.001 and a corrected cluster significance of *P* < 0.05). Significantly increased ratio relative to PD-NC in the group is shown with warm color. **(B)** Correlations between the scores of JoLO (*X*-axis) and CBF-ReHo ratio values in the left lingual gyrus (*Y*-axis). CBF, cerebral blood flow; ReHo, regional homogeneity; PD, Parkinson’s disease; NC, normal cognition; MCI, mild cognitive impairment; FWE, familywise error; JoLO, Benton’s Judgment of Line Orientation.

**FIGURE 6:**
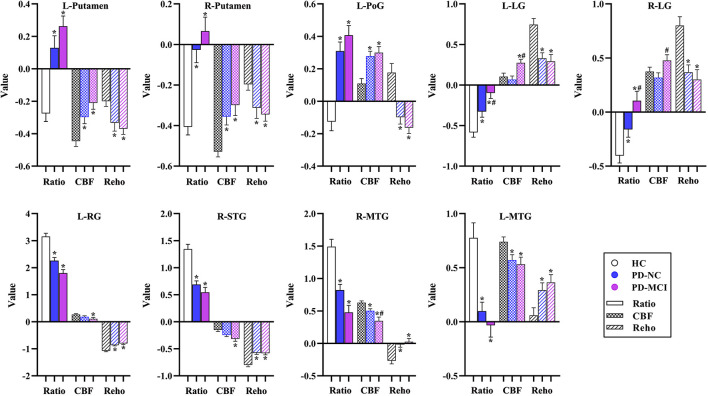
The alterations of CBF-ReHo ratio, CBF, and ReHo in brain regions with significant group differences using the voxel-based analyses. *Indicates significant intergroup difference to the HC group; ^#^indicates significant intergroup difference to the PD-NC group. CBF, cerebral blood flow; ReHo, regional homogeneity; PD, Parkinson’s disease; NC, normal cognition; MCI, mild cognitive impairment; L, left; R, right; PoG, postcentral gyrus; LG, lingual gyrus; RG, rectus gyrus; STG, superior temporal gyrus; MTG, middle temporal gyrus.

The significantly altered CBF-ReHo ratios among the three groups were distributed in the right superior temporal gyrus (STG), left rectus gyrus, bilateral middle temporal gyri (MTG), bilateral putamen, bilateral lingual gyri (LG), and left postcentral gyrus (PoG) (Figure 2). Both the PD-NC and PD-MCI groups showed higher CBF-ReHo ratios in the left LG, bilateral putamen, and left PoG and lower CBF-ReHo ratios in the right STG and bilateral MTG (Figures 3A,B) than the HC group. Despite these overlapping distributions, the PD-MCI group exhibited more extensive alterations with significantly increased CBF-ReHo ratios in the right LG and lower CBF-ReHo ratios in the bilateral parahippocampal gyri (PhGs) and right inferior frontal gyrus (IFG) (Figure 3B). Positive correlations between the mean CBF-ReHo ratio in the left putamen and UPDRS-III scores survived in both the PD-NC and PD-MCI groups (Figures 4A,B). Additionally, a higher CBF-ReHo ratio in the left LG was found in patients with PD-MCI than in patients with PD-NC (Figure 5A). Descriptions of significantly different brain regions in the CBF-ReHo ratios among groups are presented in Tables 2–4. In the PD-MCI group, the mean CBF-ReHo ratio in the left LG was negatively correlated with JoLO scores (Figure 5B). For brain regions with significant group differences in the voxel-based analyses, alterations in CBF-ReHo ratios, CBF, and ReHo are shown in Figure 6.

**TABLE 2:** Descriptions of significantly different brain regions in the CBF-ReHo ratio among groups.

Brain regions (AAL)	Peak MNI coordinates (mm)			Peak *F* value	Cluster size (mm^3^)
	X	Y	Z		
**ANOVA**					
Right superior temporal gyrus	12	9	−21	15.62	164
Left rectus gyrus	−6	36	−27	22.58	242
Left middle temporal gyrus	−63	−42	−15	11.97	54
Right middle temporal gyrus	66	−30	−9	16.56	178
Left putamen	−24	24	−9	17.95	296
Right putamen	21	24	−6	15.78	189
Left lingual gyrus	−12	−69	0	14.44	162
Right lingual gyrus	3	−66	9	9.07	61
Left postcentral gyrus	−45	−21	39	21.50	422

*These clusters are referred to multiple comparisons correction using the FWE rate (a cluster-defining threshold of *P* = 0.001 and a corrected cluster significance of *P* < 0.05).*

*CBF, cerebral blood flow; ReHo, regional homogeneity; HC, healthy control; PD, Parkinson’s disease; NC, normal cognition; MCI, mild cognitive impairment; ANOVA, analysis of variance; AAL, automated anatomical labeling; MNI, Montreal Neurological Institute; FWE, familywise error.*

**TABLE 3:** Descriptions of significantly different brain regions in the CBF-ReHo ratio between HC and PD-NC groups.

Brain regions	Peak MNI coordinates (mm)			Peak *T* value	Cluster size (mm^3^)
	X	Y	Z		
**PD-NC > HC**					
Left lingual gyrus	−12	−66	−9	4.08	49
Left putamen	−27	24	−9	4.60	85
Right putamen	36	−3	9	4.84	123
Left postcentral gyrus	−48	−18	33	5.77	237
**PD-NC < HC**					
Right superior temporal gyrus	33	15	−30	−5.80	77
Left middle temporal gyrus	−33	9	−30	−4.93	104
Right middle temporal gyrus	66	−30	−18	−4.13	61

*These clusters are referred to multiple comparisons correction using the FWE rate (a cluster-defining threshold of *P* = 0.001 and a corrected cluster significance of *P* < 0.05).*

*CBF, cerebral blood flow; ReHo, regional homogeneity; HC, healthy control; PD, Parkinson’s disease; NC, normal cognition; AAL, automated anatomical labeling; MNI, Montreal Neurological Institute; FWE, familywise error.*

**TABLE 4:** Descriptions of significantly different brain regions in the CBF-ReHo ratio between PD-MCI group and HC and PD-NC groups.

Brain regions (AAL)	Peak MNI coordinates (mm)			Peak *T* value	Cluster size (mm^3^)
	X	Y	Z		
**PD-MCI > HC**					
Left putamen	−21	21	−9	6.10	272
Right putamen	21	24	−9	5.25	170
Left lingual gyrus	−12	−69	0	5.57	158
Right lingual gyrus	15	−66	0	4.16	59
Left postcentral gyrus	−48	−21	39	5.86	348
**PD-MCI < HC**					
Right superior temporal gyrus	33	12	−45	−4.08	23
Left parahippocampal gyrus	−6	36	−27	−6.42	188
Right parahippocampal gyrus	12	9	−21	−5.46	59
Right inferior frontal gyrus	36	21	−18	−3.88	28
Left middle temporal gyrus	−63	−24	−15	−3.98	44
Right middle temporal gyrus	63	−27	−9	−5.70	173
**PD-MCI > PD-NC**					
Left lingual gyrus	−12	−78	12	3.75	26

*These clusters are referred to multiple comparisons correction using the FWE rate (a cluster-defining threshold of *P* = 0.001 and a corrected cluster significance of *P* < 0.05).*

*CBF, cerebral blood flow; ReHo, regional homogeneity; HC, healthy control; PD, Parkinson’s disease; NC, normal cognition; MCI, mild cognitive impairment; AAL, automated anatomical labeling; MNI, Montreal Neurological Institute; FWE, familywise error.*

### Distribution of Impaired Regional Homogeneity and Cerebral Blood Flow

For the analysis of ReHo maps, the significantly altered ReHo among the three groups was distributed in the right MTG, left STG, bilateral LG, and bilateral PoG. Relative to the HC subjects, higher ReHo values in right MTG and left STG were observed in patients with PD-NC, as well as higher ReHo values in the left PhG and right inferior temporal gyrus in patients with PD-MCI. Both the PD-NC and PD-MCI groups showed decreased ReHo in the bilateral LG and bilateral PoG than the HC group. There was no cluster that survived for the comparison between patients with PD-NC and PD-MCI. Descriptions of significantly different brain regions in the ReHo among groups are presented in Supplementary Tables 1–3 and Supplementary Figures 1A–C.

For the analysis of CBF maps, the significantly altered CBF among the three groups was distributed in the bilateral putamen, right insula, right MTG, left IFG, bilateral thalamus, left calcarine fissure (CF), left PoG, left paracentral lobule (PL), and right MFG. Both the PD-NC and PD-MCI groups showed higher *z*-CBF values in the bilateral putamen, bilateral thalamus, left IFG, left PoG, and left PL and lower *z*-CBF values in the right insula than the HC group. Additionally, decreased perfusion in right AG and left CF was observed in patients with PD-NC, as well as preserved perfusion in left LG and bilateral supplementary motor area and decreased perfusion in right MTG and right MFG in patients with PD-MCI. In comparison with the PD-NC group, the PD-MCI group showed higher *z*-CBF values in left CF and lower *z*-CBF values in right MTG and right MFG. Descriptions of significantly different brain regions in the CBF among groups are presented in Supplementary Tables 4–7 and Supplementary Figures 2A–D.

## Discussion

Neuroimaging characterization holds the promise of monitoring intrinsic dysfunction and revealing the corresponding pathophysiological mechanisms in PD (Rispoli et al., 2018). This study investigates neurovascular decoupling and resultant deterioration in patients with PD-MCI by integrating informative data obtained from the BOLD and ASL approaches. The PD-MCI group exhibited dysregulation of the NVU at the whole-brain GM level as well as regional disturbances in cognition-related cortices. In comparison with HC subjects, patients with PD-MCI had increased CBF-ReHo ratios in motor-related regions and decreased ratios in the limbic system and prefrontal and temporal cortices. Specifically, a distinct neurovascular decoupling in the LG that involved visuospatial dysfunction was characterized in the PD-MCI group relative to the HC and PD-NC groups. These imaging signatures linked to neurovascular coupling could potentially provide complementary insights into the underlying mechanisms of cognitive impairment during the progression of PD.

As the primary modulating element in the central nervous system, neurovascular coupling that is structurally based on the NVU ensures sufficient blood supply for neuronal activity (Yu et al., 2020). This interaction under normal physiological conditions was observed in our neuroimaging study with global and regional correlations between CBF and ReHo. Although the deleterious repercussions of neurovascular decoupling in those with PD remain poorly elucidated, recent studies have documented aberrant neuronal activity caused by dopaminergic or non-dopaminergic dysfunction as well as impaired cerebral perfusion resulting from disrupted integrity of the blood–brain barrier during the progression of PD (Ztaou et al., 2016; Dohgu et al., 2019; Henderson et al., 2020). Pathological impairments in the NVU might disrupt neurovascular coupling and consequently induce the pathogenesis underlying neurodegenerative disorders (Yan et al., 2020). Thus, our findings of compromised coordination with decreased CBF-ReHo coefficients in patients with both PD-NC and PD-MCI provide supporting evidence to the concept of neurovascular decoupling in PD, in contrast to independent alterations within each component of the NVU that have been explored by separating imaging modalities.

Guiney et al. (2015) recently proposed a mechanistic link between better neurovascular coupling and cognitive benefits. Emerging studies have concentrated on the investigation of NVU discoordination involved in pathological conditions contributing to cognitive impairment and have yielded convicting findings in AD (Liu et al., 2019), diabetes mellitus (Yan et al., 2020), and cerebrovascular diseases (Eldahshan et al., 2019). Unlike a fundamental pathway involving decoupling in these disorders, however, the underpinning mismatch responsible for the impaired cognitive profiles in PD is more complicated and accompanied by diverse pathological processes and heterogeneous symptoms. Although there was no significant intergroup difference between patients with PD-NC and PD-MCI in the global interaction, this study further explored decoupling at the regional level. Given the engagement in corresponding cognitive functions, the reduced CBF-ReHo coefficients in the MFG (executive function), the MOG (visual processing), the SMG (language processing), and the MCC, IPG, and AG, major parts of the DMN (higher-order cognitive functions), were specific to the PD-MCI group, confirming that these vulnerable regions were involved in cognitive impairment in PD from the perspective of neurovascular decoupling.

In this study, the aberrant regions of neuronal activity and cerebral perfusion in patients identified with PD-MCI were in line with those explored in previous research (Jia et al., 2018; Guo et al., 2021), although the trends in these alterations were spatially inconsistent in several regions (Figure 6). In contrast to decreased neuronal activity in motor-related regions, hypermetabolism was detected, which has been characterized as a remarkable feature of PDRP, resulting from increased energy demand (higher *z*-CBF) driven by activation of the inhibitory pathway (lower ReHo) (Chen et al., 2015). Additionally, other vasoactive mediators (i.e., dopamine and acetylcholine) or factors (i.e., neuroinflammation and oxidative stress) in PD could potentially interfere with neurovascular coupling by modulating the vascular response independent from regulation of neuronal systems (Falquetto et al., 2017; Santaella et al., 2020). Benefiting from the identification of neurovascular coupling within local regions in a voxel-wise manner, the analysis of CBF-ReHo ratios revealed comprehensive details regarding NVU changes and incongruity in patients with PD. We discovered that disproportionate interactions could be subtly reflected in CBF-ReHo ratios when alterations in the NVU were in the opposite direction. In particular, these deviations in coupling were profound within motor-related regions in patients with both PD-NC and PD-MCI. Together with the correlation between neurovascular decoupling in the putamen and disease severity, we deduced that the investigation of neurovascular decoupling is essential for the elucidation of the underlying mechanism related to movement disorders in PD. The validation of diagnostic/prognostic power for the CBF-ReHo ratio is warranted in further studies.

Divergent cognitive performances are associated with alterations in corresponding brain regions. We found deviations in the coordination in patients with PD-MCI with decreased ratios in the limbic system (STG and PhG) and prefrontal (IFG) and temporal (MTG) cortices, which corresponds well with the vulnerable regions that have been closely implicated in cognitive dysfunction (Adler and Beach, 2016). Importantly, intergroup differences in the LG with a higher CBF-ReHo ratio were identified in this study. As the core hub that manages visual processing function, the LG has been reported to show aberrant functional, metabolic, and structural alterations, contributing to cognitive decline in patients with PD (Kamagata et al., 2011; Lee et al., 2013; Wang et al., 2018). Given the dual syndrome hypothesis proposed within the neuropathological literature (Kehagia et al., 2013), we hypothesized that decoupling in the LG was mainly subtended by cholinergic dysfunction rather than nigrostriatal degradation. Longitudinal studies have implied that impairments in visuospatial processing in patients with PD are superior to declines in episodic memory for the prediction of progression to dementia (Williams-Gray et al., 2009; Wolters et al., 2019). Although the precise pathogenic substrates cannot be distinguished by CBF-ReHo ratios, our correlation analysis demonstrated that decoupling in the LG was associated with poor visual–spatial performance scored using the JoLO scale, confirming that this integrated metric of neurovascular coupling could function as a promising imaging biomarker of cognitive decline in patients with PD-MCI.

Several limitations of this study should be mentioned. First, the sample size in this study was relatively small, which might have influenced the statistical analysis. Further longitudinal investigations are required with a larger cohort including individuals with dementia as well as stricter head motion correction. Second, the elucidation of specific neurovascular mechanisms underlying PD-MCI still needs in-depth exploration that directly uses advanced imaging modalities or pathophysiological models. Third, although neuropsychological scales were employed for each cognitive domain, more detailed scales are recommended for the comprehensive understanding of corresponding cognitive performance. Finally, the clinical and MRI data were obtained on the “OFF” medication state to mitigate pharmacological effects. However, medication-free patients are required in future studies to validate our findings.

## Conclusion

In summary, this study found that patients with PD-MCI show neurovascular decoupling associated with deteriorated motor function and cognitive performance. Most prominently, the LG in patients with PD-MCI showed region-specific effects associated with visual–spatial function characterized by NVU dysfunction, i.e., disrupted CBF-ReHo correlation coefficients and CBF-ReHo ratios. Our research illuminated the involvement of neurovascular decoupling in the neurodegenerative process in PD-MCI and emphasized the potential role of coupling metrics for the mechanistic investigation of the cognitive decline associated with PD.

## Data Availability Statement

The original contributions presented in the study are included in the article/Supplementary Material, further inquiries can be directed to the corresponding authors.

## Ethics Statement

The studies involving human participants were reviewed and approved by the Research Ethics Committee of Nanjing Medical University. The patients/participants provided their written informed consent to participate in this study.

## Author Contributions

SS, HZ, and YF made substantial contributions to the conception and design of this study. SS, JW, and WD made substantial contributions to the acquisition of data. SS, HZ, and Y-CC made substantial contributions to the analysis of data. SS, YF, Y-CC, and XY contributed to the interpretations of data. SS and HZ drafted the first version of the manuscript. All authors revised the draft for intellectual content, gave their final approval of the final version for publication, and agreed to be accountable for all aspects of the work in ensuring that questions related to the accuracy or integrity of any part of this study were appropriately investigated and resolved.

## Conflict of Interest

The authors declare that the research was conducted in the absence of any commercial or financial relationships that could be construed as a potential conflict of interest.

## Publisher’s Note

All claims expressed in this article are solely those of the authors and do not necessarily represent those of their affiliated organizations, or those of the publisher, the editors and the reviewers. Any product that may be evaluated in this article, or claim that may be made by its manufacturer, is not guaranteed or endorsed by the publisher.

## Footnotes

1
http://rfmri.org/dpabi

2
http://www.fil.ion.ucl.ac.uk/spm

## Supplementary Material

Supplementary Figure 1 The brain regions with significant differences of ReHo among groups. The voxel-based analysis demonstrates the survived clusters among HC, PD-NC, and PD-MCI groups **(A)**, between HC and PD-NC groups **(B)**, and between HC and PD-MCI groups **(C)**. These clusters are referred to multiple comparisons correction using the FWE rate (a cluster-defining threshold of *P* = 0.001 and a corrected cluster significance of *P* < 0.05). The region with the significant difference among the three groups is shown with the warm color (for **A**). The significantly increased ratio relative to HC in the group is shown with warm color, and the significantly decreased ratio relative to HC in the group is shown with cold color (for **B,C**). ReHo, regional homogeneity; HC, healthy control; PD, Parkinson’s disease; NC, normal cognition; MCI, mild cognitive impairment; FWE, familywise error; R, right; L, left.

Supplementary Figure 2 The brain regions with significant differences of CBF among groups. The voxel-based analysis demonstrates the survived clusters among HC, PD-NC, and PD-MCI groups **(A)**, between HC and PD-NC groups **(B)**, between HC and PD-MCI groups **(C)**, and between PD-NC and PD-MCI groups **(D)**. These clusters are referred to multiple comparisons correction using the FWE rate (a cluster-defining threshold of *P* = 0.001 and a corrected cluster significance of *P* < 0.05). The region with the significant difference among the three groups is shown with the warm color (for **A**). The significantly increased ratio relative to HC in the group is shown with warm color, and the significantly decreased ratio relative to HC in the group is shown with cold color (for **B,C**). The significantly increased ratio relative to PD-NC in the group is shown with warm color, and the significantly decreased ratio relative to PD-NC in the group is shown with cold color (for **D**). CBF, cerebral blood flow; HC, healthy control; PD, Parkinson’s disease; NC, normal cognition; MCI, mild cognitive impairment; FWE, familywise error; R, right; L, left.
