# Supplementary material for: Region-Specific Neurovascular Decoupling Associated With Cognitive Decline in Parkinson’s Disease
Source: Front Aging Neurosci. 2021 Nov 15;13:770528. doi: 10.3389/fnagi.2021.770528 (PMC8636132; doi:10.3389/fnagi.2021.770528)
Supplement: Supplementary file 1 [file Table_1.pdf]

Table S1. Descriptions of significantly different brain regions in ReHo among groups

Brain regions (AAL)	Peak MNI coordinates (mm)			Peak F value	Cluster size (mm^3)
	X	Y	Z		
ANOVA					
Right middle temporal gyrus	24	15	-36	15.39	166
Left superior temporal gyrus	-12	21	-27	25.38	558
Bilateral lingual gyri	-9	-66	6	13.05	295
Right postcentral gyrus	48	-15	36	15.04	128
Left postcentral gyrus	-45	-21	39	17.25	340

These clusters are referred to multiple comparisons correction using the FWE rate (a cluster-defining threshold of $P = 0.001$ and a corrected cluster significance of $P < 0.05$).

Abbreviations: ReHo, regional homogeneity; ANOVA, one-way analysis of variance; AAL, automated anatomical labeling; MNI, Montreal Neurological Institute; FWE, familywise error

Table S2. Descriptions of significantly different brain regions in ReHo between HC and PD-NC groups

Brain regions	Peak MNI coordinates (mm)			Peak T value	Cluster size (mm^3)
	X	Y	Z		
**PD-NC > HC**					
Right middle temporal gyrus	27	15	-36	5.06	144
Left superior temporal gyrus	-15	21	-27	6.16	251
**PD-NC < HC**					
Bilateral lingual gyri	15	-54	-3	-4.65	250
Right postcentral gyrus	48	-15	36	-5.53	113
Left postcentral gyrus	-45	-18	39	-5.15	122

These clusters are referred to multiple comparisons correction using the FWE rate (a cluster-defining threshold of $P = 0.001$ and a corrected cluster significance of $P < 0.05$).

Abbreviations: ReHo, regional homogeneity; HC, healthy control; PD, Parkinson's disease; NC, normal cognition; AAL, automated anatomical labeling; MNI, Montreal Neurological Institute; FWE, familywise error

Table S3. Descriptions of significantly different brain regions in ReHo between PD-MCI and HC groups

Brain regions (AAL)	Peak MNI coordinates (mm)			Peak T value	Cluster size (mm^3)
	X	Y	Z		
**PD-MCI > HC**					
Left parahippocampal gyrus	-6	30	-27	6.95	530
Right inferior temporal gyrus	30	-15	-33	4.69	88
**PD-MCI < HC**					
Bilateral lingual gyri	3	-66	6	-4.43	208
Right postcentral gyrus	60	-6	33	-4.25	76
Left postcentral gyrus	-51	-18	-51	-5.05	311

These clusters are referred to multiple comparisons correction using the FWE rate (a cluster-defining threshold of $P = 0.001$ and a corrected cluster significance of $P < 0.05$).

Abbreviations: ReHo, regional homogeneity; HC, healthy control; PD, Parkinson's disease; MCI, mild cognitive impairment; AAL, automated anatomical labeling; MNI, Montreal Neurological Institute; FWE, familywise error

Table S4. Descriptions of significantly different brain regions in CBF among groups

Brain regions (AAL)	Peak MNI coordinates (mm)			Peak F value	Cluster size (mm^3)
	X	Y	Z		
**ANOVA**					
Left putamen	-33	-3	3	23.63	266
Right insula	48	12	-6	15.87	181
Right middle temporal gyrus	69	-30	-6	17.02	341
Left inferior frontal gyrus	-24	42	-9	21.66	148
Right thalamus	15	-18	9	28.90	114
Left calcarine fissure	-12	-48	9	11.80	156
Left thalamus	-15	-21	6	22.06	98
Right putamen	33	-3	6	15.07	90
Left postcentral gyrus	-57	-15	21	19.62	102
Left paracentral lobule	-3	-27	63	13.42	233
Right middle frontal gyrus	33	24	51	17.00	101

These clusters are referred to multiple comparisons correction using the FWE rate (a cluster-defining threshold of $P = 0.001$ and a corrected cluster significance of $P < 0.05$).

Abbreviations: CBF, cerebral blood flow; ANOVA, one-way analysis of variance; AAL, automated anatomical labeling; MNI, Montreal Neurological Institute

Table S5. Descriptions of significantly different brain regions in CBF between HC and PD-NC groups

Brain regions	Peak MNI coordinates (mm)			Peak T value	Cluster size (mm^3)
	X	Y	Z		
**PD-NC > HC**					
Left putamen	-33	-3	3	5.44	158
Right thalamus	15	-18	9	7.22	94
Right putamen	33	0	3	4.88	84
Left thalamus	-12	-18	6	5.38	63
Left inferior frontal gyrus	-36	33	3	4.29	26
Left postcentral gyrus	-57	-15	24	4.83	58
Left paracentral lobule	-3	-27	63	4.93	199
**PD-NC < HC**					
Right insula	48	12	-6	-4.95	115
Right angular gyrus	60	-54	30	-5.56	133
Left calcarine fissure	-21	-60	24	-4.13	41

These clusters are referred to multiple comparisons correction using the FWE rate (a cluster-defining threshold of $P = 0.001$ and a corrected cluster significance of $P < 0.05$).

Abbreviations: CBF, cerebral blood flow; HC, healthy control; PD, Parkinson's disease; NC, normal cognition; AAL, automated anatomical labeling; MNI, Montreal Neurological Institute; FWE, familywise error

Table S6. Descriptions of significantly different brain regions in CBF between PD-MCI and HC groups

Brain regions	Peak MNI coordinates (mm)			Peak T value	Cluster size (mm^3)
	X	Y	Z		
**PD-MCI > HC**					
Left putamen	-33	-3	6	6.26	231
Left inferior frontal gyrus	-33	33	6	5.17	135
Right thalamus	18	-18	12	6.11	101
Right putamen	21	18	3	5.23	75
Left lingual gyrus	-15	-42	-9	4.56	35
Left thalamus	-15	-21	6	5.66	98
Left postcentral gyrus	-57	-15	21	5.40	99
Left supplementary motor area	-15	-12	48	4.90	57
Right supplementary motor area	9	-9	51	4.14	34
Left paracentral lobule	-3	-27	54	4.73	78
**PD-MCI < HC**					
Right insula	48	18	0	-5.03	163
Right middle temporal gyrus	66	-24	-6	-5.60	303
Right middle frontal gyrus	33	24	51	-5.97	101

These clusters are referred to multiple comparisons correction using the FWE rate (a cluster-defining threshold of $P = 0.001$ and a corrected cluster significance of $P < 0.05$).

Abbreviations: CBF, cerebral blood flow; HC, healthy control; PD, Parkinson's disease; MCI, mild cognitive impairment; AAL, automated anatomical labeling; MNI, Montreal Neurological Institute; FWE, familywise error

Table S7. Descriptions of significantly different brain regions in CBF between PD-NC and PD-MCI groups

Brain regions	Peak MNI coordinates (mm)			Peak T	Cluster size
	X	Y	Z	value	(mm^3)
**PD-MCI > PD-NC**					
Left calcarine fissure	-12	-69	9	4.59	134
**PD-MCI < PD-NC**					
Right middle temporal gyrus	69	-27	3	-4.47	32
Right middle frontal gyrus	33	24	51	-4.66	34

These clusters are referred to multiple comparisons correction using the FWE rate (a cluster-defining threshold of $P = 0.001$ and a corrected cluster significance of $P < 0.05$).

Abbreviations: CBF, cerebral blood flow; HC, healthy control; PD, Parkinson's disease; NC, normal cognition; MCI, mild cognitive impairment; AAL, automated anatomical labeling; MNI, Montreal Neurological Institute; FWE, familywise error
