# Supplementary figures and images for: Region-Specific Neurovascular Decoupling Associated With Cognitive Decline in Parkinson’s Disease
Source: Front Aging Neurosci. 2021 Nov 15;13:770528. doi: 10.3389/fnagi.2021.770528 (PMC8636132; doi:10.3389/fnagi.2021.770528)

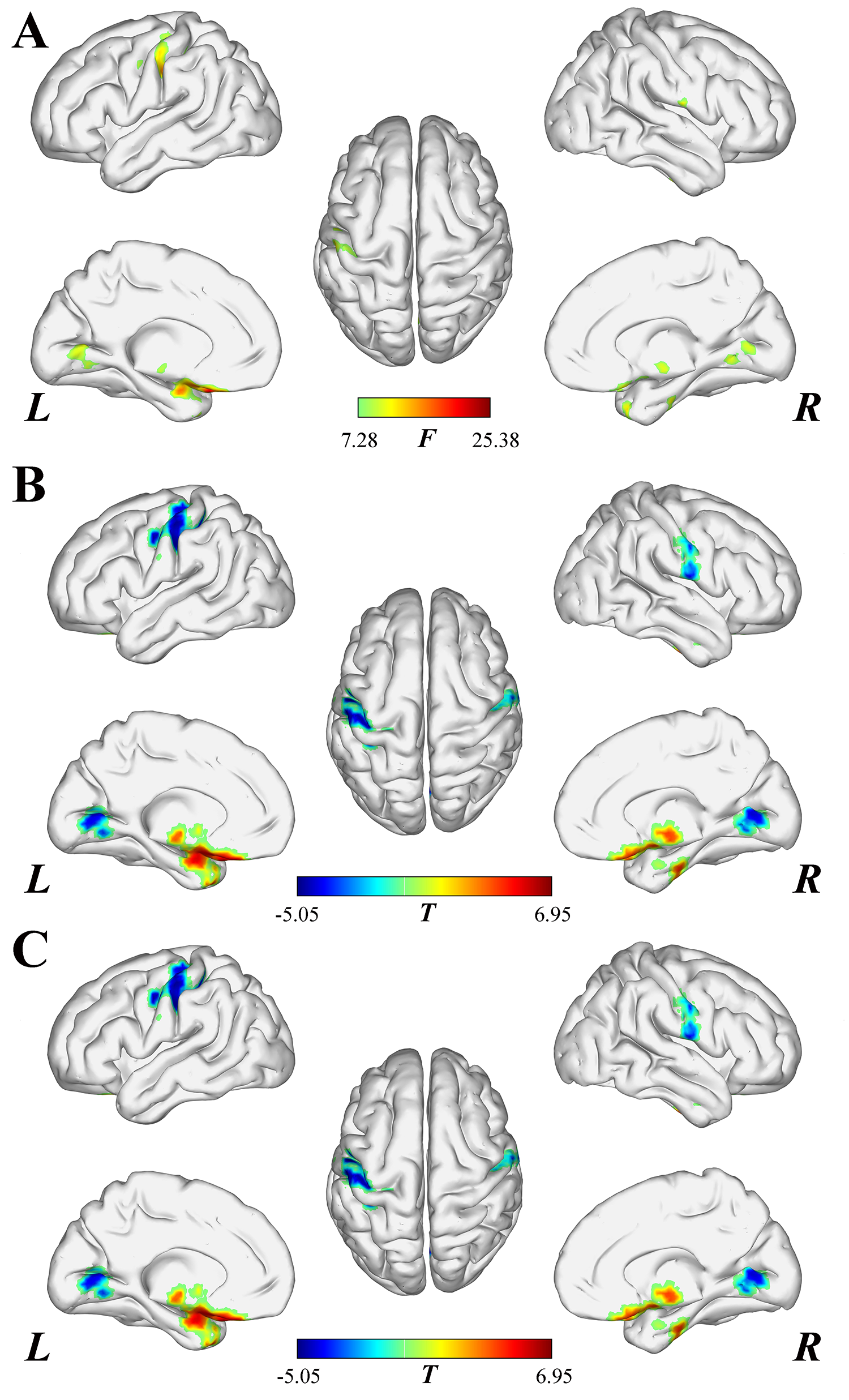

Supplement: Supplementary Figure 1 — The brain regions with significant differences of ReHo among groups. The voxel-based analysis demonstrates the survived clusters among HC, PD-NC, and PD-MCI groups (A), between HC and PD-NC groups (B), and between HC and PD-MCI groups (C). These clusters are referred to multiple comparisons correction using the FWE rate (a cluster-defining threshold of P = 0.001 and a corrected cluster significance of P < 0.05). The region with the significant difference among the three groups is shown with the warm color (for A). The significantly increased ratio relative to HC in the group is shown with warm color, and the significantly decreased ratio relative to HC in the group is shown with cold color (for B,C). ReHo, regional homogeneity; HC, healthy control; PD, Parkinson’s disease; NC, normal cognition; MCI, mild cognitive impairment; FWE, familywise error; R, right; L, left. [file Image_1.TIF]

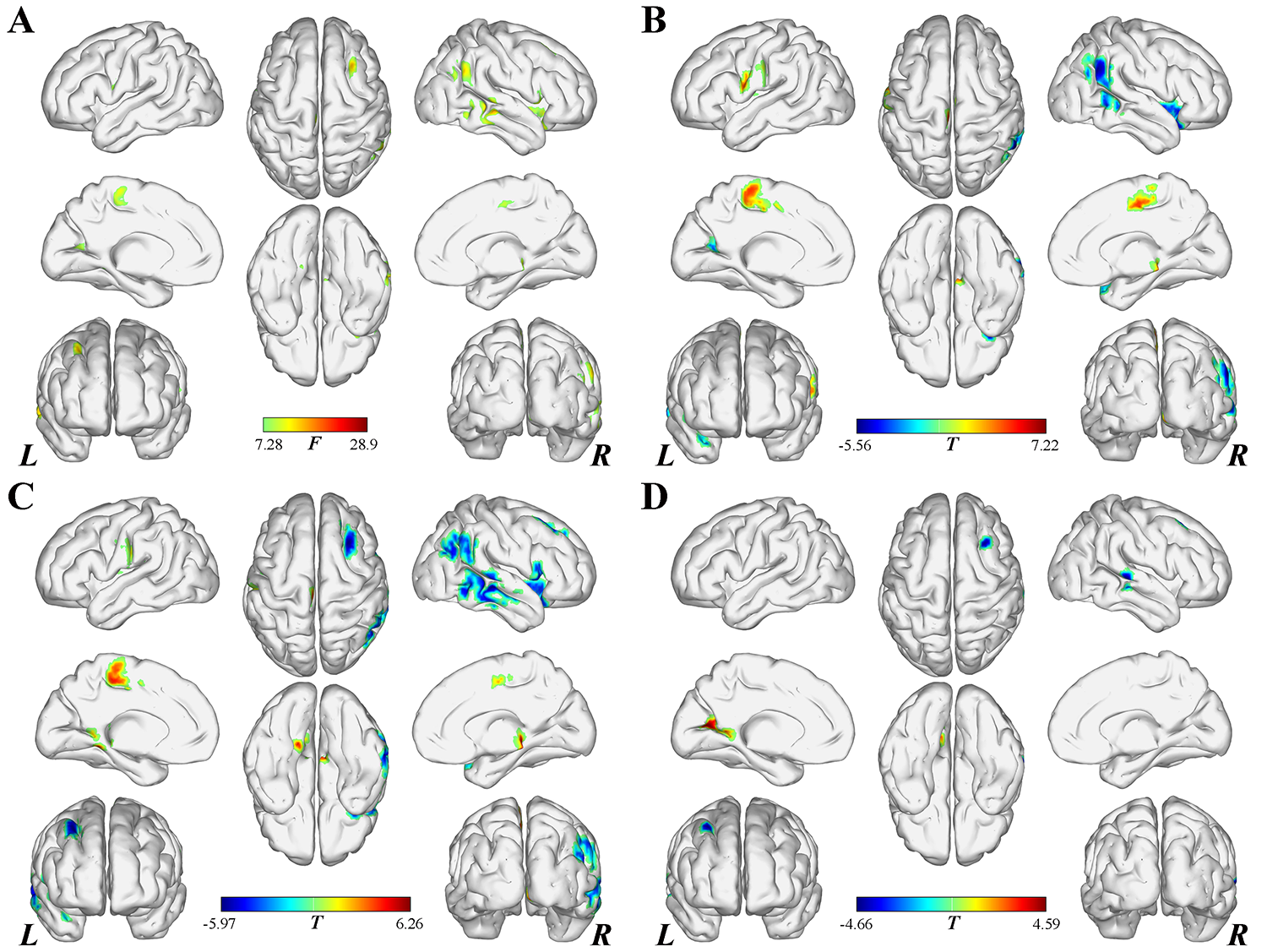

Supplement: Supplementary Figure 2 — The brain regions with significant differences of CBF among groups. The voxel-based analysis demonstrates the survived clusters among HC, PD-NC, and PD-MCI groups (A), between HC and PD-NC groups (B), between HC and PD-MCI groups (C), and between PD-NC and PD-MCI groups (D). These clusters are referred to multiple comparisons correction using the FWE rate (a cluster-defining threshold of P = 0.001 and a corrected cluster significance of P < 0.05). The region with the significant difference among the three groups is shown with the warm color (for A). The significantly increased ratio relative to HC in the group is shown with warm color, and the significantly decreased ratio relative to HC in the group is shown with cold color (for B,C). The significantly increased ratio relative to PD-NC in the group is shown with warm color, and the significantly decreased ratio relative to PD-NC in the group is shown with cold color (for D). CBF, cerebral blood flow; HC, healthy control; PD, Parkinson’s disease; NC, normal cognition; MCI, mild cognitive impairment; FWE, familywise error; R, right; L, left. [file Image_2.TIF]
